# Protein Malnutrition Modifies Innate Immunity and Gene Expression by Intestinal Epithelial Cells and Human Rotavirus Infection in Neonatal Gnotobiotic Pigs

**DOI:** 10.1128/mSphere.00046-17

**Published:** 2017-03-01

**Authors:** Anastasia N. Vlasova, Francine C. Paim, Sukumar Kandasamy, Moyasar A. Alhamo, David D. Fischer, Stephanie N. Langel, Loic Deblais, Anand Kumar, Juliet Chepngeno, Lulu Shao, Huang-Chi Huang, Rosario A. Candelero-Rueda, Gireesh Rajashekara, Linda J. Saif

**Affiliations:** Food Animal Health Research Program (FAHRP), Ohio Agricultural Research and Development Center, Veterinary Preventive Medicine Department, The Ohio State University, Wooster, Ohio, USA; Virginia Tech

**Keywords:** intestinal epithelial cells, gnotobiotic piglets, human infant fecal microbiota, human rotavirus, innate immunity, intestinal epithelial barrier, natural killer cells, plasmacytoid dendritic cells, protein deficiency

## Abstract

Malnutrition affects millions of children in developing countries, compromising immunity and contributing to increased rates of death from infectious diseases. Rotavirus is a major etiological agent of childhood diarrhea in developing countries, where malnutrition is prevalent. However, the interactions between the two and their combined effects on immune and intestinal functions are poorly understood. In this study, we used neonatal gnotobiotic (Gn) pigs transplanted with the fecal microbiota of a healthy 2-month-old infant (HIFM) and fed protein-deficient or -sufficient bovine milk diets. Protein deficiency induced hypoproteinemia, hypoalbuminemia, hypoglycemia, stunting, and generalized edema in Gn pigs, as observed in protein-malnourished children. Irrespective of the diet, human rotavirus (HRV) infection early, at HIFM posttransplantation day 3 (PTD3), resulted in adverse health effects and higher mortality rates (45 to 75%) than later HRV infection (PTD10). Protein malnutrition exacerbated HRV infection and affected the morphology and function of the small intestinal epithelial barrier. In pigs infected with HRV at PTD10, there was a uniform decrease in the function and/or frequencies of natural killer cells, plasmacytoid dendritic cells, and CD103^+^ and apoptotic mononuclear cells and altered gene expression profiles of intestinal epithelial cells (chromogranin A, mucin 2, proliferating cell nuclear antigen, SRY-Box 9, and villin). Thus, we have established the first HIFM-transplanted neonatal pig model that recapitulates major aspects of protein malnutrition in children and can be used to evaluate physiologically relevant interventions. Our findings provide an explanation of why nutrient-rich diets alone may lack efficacy in malnourished children.

**IMPORTANCE** Malnutrition and rotavirus infection, prevalent in developing countries, individually and in combination, affect the health of millions of children, compromising their immunity and increasing the rates of death from infectious diseases. However, the interactions between the two and their combined effects on immune and intestinal functions are poorly understood. We have established the first human infant microbiota-transplanted neonatal pig model of childhood malnutrition that reproduced the impaired immune, intestinal, and other physiological functions seen in malnourished children. This model can be used to evaluate relevant dietary and other health-promoting interventions. Our findings provide an explanation of why adequate nutrition alone may lack efficacy in malnourished children.

## Abstract (precis)

Malnutrition and rotavirus infection, prevalent in developing countries, individually and in combination, affect the health of millions of children, compromising their immunity and increasing the rates of death from infectious diseases. However, the interactions between the two and their combined effects on immune and intestinal functions are poorly understood. We have established the first human infant microbiota-transplanted neonatal pig model of childhood malnutrition that reproduced the impaired immune, intestinal, and other physiological functions seen in malnourished children. This model can be used to evaluate relevant dietary and other health-promoting interventions. Our findings provide an explanation of why adequate nutrition alone may lack efficacy in malnourished children.

## INTRODUCTION

Malnutrition, alone or combined with enteric infections, contributes to almost half of all deaths of children under 5 years old (1). Initiated by protein deprivation, the ensuing intestinal dysbiosis, epithelial breaches, altered metabolism, and compromised immune system (1, 2) promote pathogen/pathobiont invasion, leading to environmental enteric dysfunction (EED) in malnourished children (3). The resulting persistent diarrhea remains the second most common cause of childhood death in the developing world. The multifactorial pathobiology of childhood malnutrition and the associated EED is best described as a vicious cycle of impaired immunity, recurrent enteric infections, intestinal inflammation, and malabsorption that, in turn, worsens malnutrition. Protein deficiency and dysbiotic intestinal microbiota are major contributors to kwashiorkor (clinically manifested by stunting and edema) and “hidden hunger” or micronutrient deficiencies, further compromising immunity and the epithelial barrier (1). The self-renewal and differentiation of intestinal epithelial stem cells (IESCs) regulate intestinal epithelial cell (IEC) homeostasis and turnover (4). Childhood protein deficiency impairs IESC functions, compromising the host’s ability to repair the intestinal epithelium upon intestinal injury or infection (5). Therefore, IESC dysfunction may represent one of the key mechanisms in the pathophysiology of malnutrition and EED. Historically, childhood malnutrition studies have focused on understanding its effects on health, metabolism, and susceptibility to enteric or respiratory infections. Rodent models, commonly used to study single nutrient deficiencies, do not recapitulate all of the clinical, immunological, and molecular parameters of malnutrition and are of uncertain relevance in the modeling of the increased infection rates seen in malnourished human populations (6). Studies of Malawian twins discordant for kwashiorkor in Gn mouse/pig models demonstrated a causal microbiota-dependent relationship between mother-derived nutrients, including milk oligosaccharides, and neonatal growth, nutritional status, metabolism, and organ morphology (7, 8).

Most recent estimates show that rotavirus (RV) diarrhea claims the lives of ~215,000 children under age 5 years old annually worldwide (9, 10). Although there are inconsistent findings on the interactions between RV and malnutrition (11–14), increased human RV (HRV) disease severity and vaccine failures (15) in developing countries are generally associated with reduced immune function stemming from intestinal dysbiosis, nutrient deficiencies, and environmental enteropathy. However, contradictory evidence comes from a longitudinal study of a birth cohort in Bangladesh that demonstrated a positive association between healthy dwelling and adequate nutritional status of infants with the risk of symptomatic RV infection (16). This observation may be related to the preexisting damage of the intestinal epithelium of infants who had clinical or subclinical malnutrition and may therefore lack the target mature enterocytes that support HRV infection. These studies necessitate further research to understand the complex molecular mechanisms of the interactions among malnutrition, immune function, and intestinal homeostasis.

Here we established a human infant fecal microbiota (HIFM)-transplanted neonatal gnotobiotic (Gn) pig model of childhood malnutrition and investigated the impacts of early-life protein malnutrition on innate immunity, pathogenesis of an enteric infection (HRV), and IEC gene expression (chromogranin A [CgA], enteroendocrine cells; mucin 2 [MUC2], goblet cells; proliferating cell nuclear antigen [PCNA], transient amplifying progenitor cells; SRY-Box 9 [SOX9], IESCs; villin, enterocytes).

## RESULTS

### Protein deficiency produces failure to thrive in neonatal piglets.

**FIG 1:**
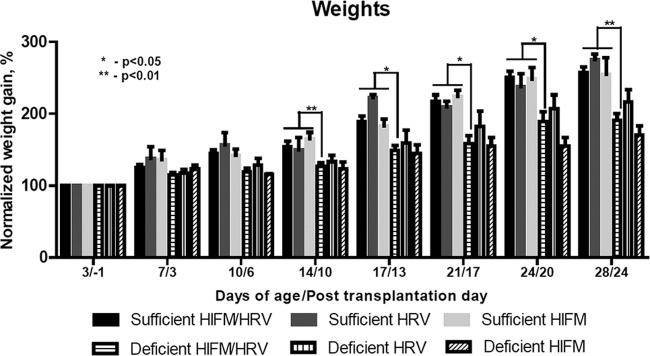
Normalized weight gain of protein-sufficient and -deficient pigs after HRV inoculation and HIFM transplantation monitored over a period of 4 weeks. The results are the mean ± SEM from 10 to 27 animals in the deficient- and sufficient-diet HIFM/HRV groups (*n* = 17 to 27 for early [prior to day of age 10] time points; *n* = 10 for later time points), the results of the early and late HRV/HIFM groups were combined because there were no differences in the weights due to the timing of HRV infection; five or six animals in the deficient- and sufficient-diet HRV groups, and two or three animals in the deficient- and sufficient-diet HIFM groups. The results shown are the mean ± SEM. Difference in stunting from the sufficient-diet counterpart (two-way ANOVA): *, significant (*P* ≤ 0.05); **, highly significant (*P* ≤ 0.01).

**FIG 2:**
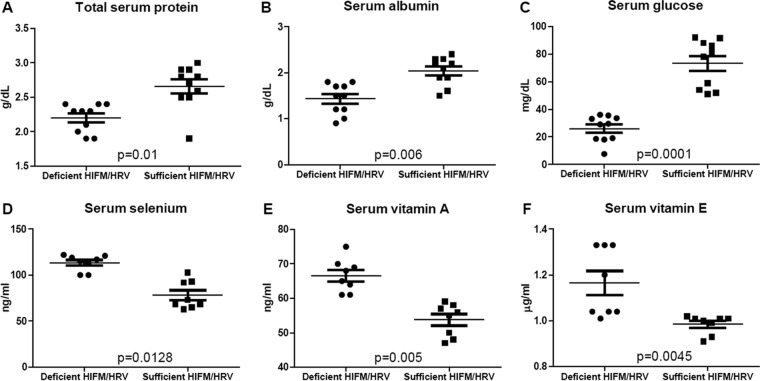
Concentrations of protein (A), albumin (B), glucose (C), selenium (D), vitamin A (E), and vitamin E (F) in the serum of protein-deficient and -sufficient HIFM/HRV-infected pigs measured at experiment termination. Concentrations of protein, albumin, and glucose in the serum of protein-deficient and -sufficient HIFM/HRV-infected pigs (*n* = 10/group) were determined at Marshfield Labs (Marshfield, WI), and selenium, vitamin A and E concentrations in the serum of protein-deficient and -sufficient HIFM/HRV-infected pigs (*n* = 8/group) were measured at the Diagnostic Center for Population and Animal Health (Michigan State University, East Lansing, MI) by high-performance liquid chromatography. The normal ranges of the levels of these elements in the plasma or serum of conventional suckling piglets are 44 to 91 mg/dl for glucose, 5.8 to 8.1 g/dl for protein, 3.2 to 4.2 g/dl for albumin, 100 to 350 ng/ml for vitamin A, 0.4 to 5 μg/ml for vitamin E, and 50 to 150 ng/ml for selenium; however, the respective ranges in Gn pigs are not established. Concentrations were analyzed with the Kruskal-Wallis rank sum test. The results shown are the mean ± SEM.

At 1 week of age, stunting (decreased or no weight gain) occurred in all of the pigs in the protein-deficient groups; at 2 weeks and thereafter, it was highly statistically significant for HIFM-transplanted HRV-inoculated pigs (Fig. 1). Edema of the face, neck, feet, and abdominal area (commonly observed in human cases of kwashiorkor) developed at 10 days of age and persisted throughout the duration of the experiments in protein-deficient pigs but not in protein-sufficient pigs. Comparison of blood parameters at the termination of the experiment revealed that pigs on the protein-deficient diet had significantly decreased levels of glucose, total protein, and albumin (Fig. 2, HIFM/HRV-infected pigs on deficient and sufficient diets; no-HRV/no-HIFM controls, data not shown), as seen in malnourished children (which develop because of malnutrition itself and malabsorption associated with malnutrition). However, these trends were opposite early after HRV inoculation (postinfection day 2 [PID2]) (data not shown). Additionally, serum vitamin A, vitamin E, and selenium levels were significantly increased at the end of the experiment in protein-deficient groups but not in protein-sufficient groups, suggestive of a positive “30-day response” (as seen in vitamin A-deficient children [17]) to supplementation of these micronutrients in the diet. The mortality rate of the HIFM-transplanted piglets on a protein-sufficient diet (no HRV) did not exceed 14% (1 piglet/litter, on average), whereas the mortality rates of the HIFM-transplanted piglets (no HRV) on the protein-deficient diet ranged between 15 and 45% (Table 1). The clinical signs in the affected piglets were consistent with hypoglycemia and septicemia in addition to extraintestinal (liver, kidney, lung, and peritoneal cavity swabs) recovery of *Clostridium perfringens* type A and nonhemolytic, avirulent *Escherichia coli* (bacterial identification to the species level was conducted at the Ohio Department of Agriculture).

**TABLE 1:** Mortality rates of pigs in different experimental groups

**Exptl group**	**Mortality rate (%)**^a^
Deficient/HIFM/early HRV	*45–75*
Sufficient/HIFM/early HRV	
Deficient/HIFM/late HRV	**15–45**
Sufficient/HIFM/late HRV	0–14
Deficient/late HRV	**15–33**
Sufficient/late HRV	0
Deficient/HIFM	**15–45**
Sufficient/HIFM	0–14

a Italics indicate that early HRV inoculation of HIFM-transplanted pigs resulted in mortality rates significantly higher (*P* = 0.0001) than those of all of the other experimental groups, irrespective of the diet. Bold values indicate statistically significant higher (*P* = 0.0002 to 0.0047) mortality rates of deficient-diet pigs than their sufficient-diet counterparts.

### Early HRV infection results in adverse health effects and increases the mortality rate in neonatal HIFM-transplanted Gn pigs.

**FIG 3:**
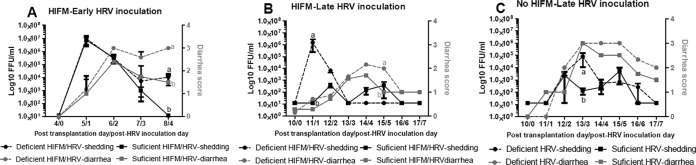
Diarrhea severity and fecal HRV shedding in protein-deficient and -sufficient HIFM/HRV-infected pigs. Virus shedding was determined by cell culture immunofluorescence assay and is expressed as log_10_ FFU/ml (left *y* axis), and diarrhea severity was determined by fecal consistency >1 (right *y* axis [fecal consistency was scored as follows: 0, normal; 1, pasty/semiliquid; 2, liquid; 3, watery]): in HIFM-transplanted pigs after early (PTD3, *n* = 5 to 8/group) (A) and late (PTD10, *n* = 10/group) (B) HRV infection and in GF pigs after late (PTD10, *n* = 5 or 6/group) HRV infection (C). Different letters (a and b) indicate significant differences (two-way ANOVA, *P* ≤ 0.05) among treatment groups at the same time point. The results shown are the mean ± SEM.

In pilot experiments, to evaluate the effects of protein malnutrition on immune function and enteric infection, we infected pigs with HRV at 3 (early) and 10 (late) days after HIFM transplantation to evaluate protein malnutrition/HRV infection effects in the context of developing and established intestinal microbiomes, respectively. The neonatal pigs infected with HRV at 3 days after HIFM transplantation had poor health (weight loss, lethargy, neurological signs including ataxia, diarrhea, a moribund state, loss of appetite, and transient fever), high mortality rates (45 to 75%, Table 1), and enhanced HRV shedding/diarrhea, unlike pigs infected at 10 days after HIFM transplantation or germfree (GF) pigs (Fig. 3). Although fecal HRV shedding was comparable at PID0 to PID2, it was higher in deficient pigs thereafter following early HRV infection (Fig. 3A). Similarly, diarrhea was more severe and persisted in deficient pigs through experiment termination at PID5 (Fig. 3A). By PID5 after early HRV infection, most (75%) of the pigs died and the surviving ones failed to thrive or had recurrent fever or mild neurological symptoms, irrespective of the diet, and were euthanized. Comparison of early immunological parameters did not reveal consistent trends. Therefore, to avoid the adverse reactions and an excessively high mortality rate, all of the pigs in the subsequent experiments were inoculated with HRV at 10 days after HIFM transplantation.

### HRV infection (diarrhea, shedding) is more severe in protein-deficient piglets after late HRV inoculation.

We conducted several trials to compare immune responses, HRV pathogenesis, and IEC gene expression in protein-sufficient and -deficient pigs after HRV inoculation at 10 days after HIFM transplantation. Additionally, we included groups of GF (no HIFM transplantation) pigs inoculated with HRV and HIFM-transplanted pigs without HRV inoculation on protein-sufficient and -deficient diets to evaluate HIFM/HRV contributions to clinical signs of malnutrition. The GF pigs on the protein-deficient diet became stunted compared to their sufficient-diet counterparts; however, stunting (Fig. 1) and edema (by appearance) were less evident in GF pigs than in HIFM-transplanted protein-deficient pigs. HIFM-transplanted pigs on the protein-deficient diet but without HRV inoculation became stunted similarly to their HRV-infected counterparts (Fig. 1).

HRV shedding and diarrhea were increased in deficient- versus sufficient-diet pigs (Fig. 3B and C). A significantly higher first peak of fecal HRV shedding was observed in deficient-diet HIFM pigs; however, they lacked the second peak of shedding present in the sufficient-diet group (Fig. 3B and C). Additionally, while the diarrhea scores of GF pigs were higher, their HRV shedding titers were comparable to those of HIFM-transplanted pigs (Fig. 3), suggesting that the commensal microbiota decreases the severity of HRV diarrhea.

### Protein deficiency affects the intestinal epithelial barrier.

**FIG 4:**
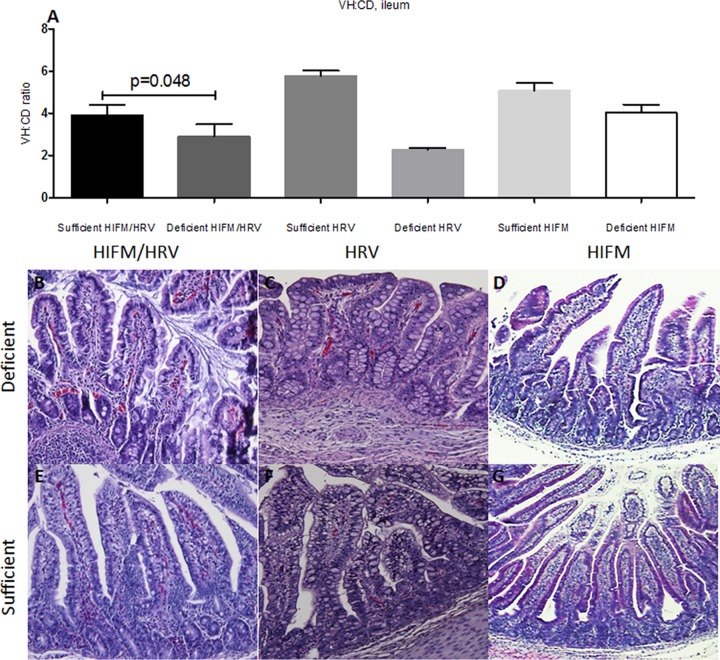
Evaluation of VH/CD ratios of ileal villi of protein-deficient (A to D) and -sufficient (A and E to G) pigs by H&E staining. Ileal sections of pigs euthanized at PID14/PTD24 were evaluated. Mean VH/CD ratios of different groups are shown in the graph in panel A (*n =* 2 or 3 for sufficient- and deficient-diet HIFM or HRV-infected pigs, and *n =* 5 to 10 for sufficient- and deficient-diet HIFM or HRV-infected pigs), and representative images for deficient- and sufficient-diet HIFM/HRV, deficient- and sufficient-diet HRV, and deficient- and sufficient-diet HIFM groups are shown in panels B and E, C and F, and D and G, respectively. The results shown are the mean ± SEM. *P* < 0.05 was considered statistically significant by the Mann-Whitney test.

**FIG 5:**
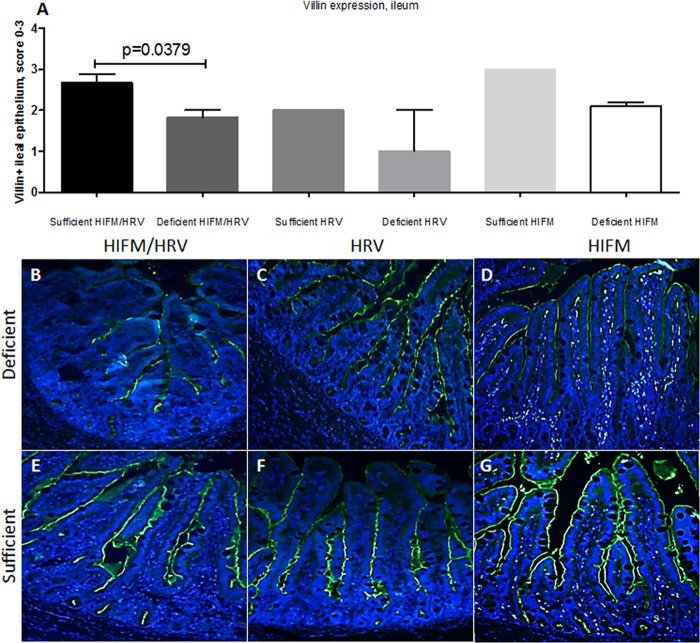
Villin expression by IECs of protein-deficient (A to D) and -sufficient (A and E to G) pigs assessed by immunofluorescent staining. Ileal sections of pigs euthanized at PID14/PTD24 were evaluated. The mean villin-positive scores of different groups are shown in graph A (*n =* 2 or 3 for sufficient and deficient HIFM or HRV-infected pigs; *n =* 5 to 10 for sufficient- and deficient-diet HIFM or HRV-infected pigs), and representative images for the deficient- and sufficient-diet HIFM/HRV, deficient- and sufficient-diet HRV, and deficient- and sufficient-diet HIFM groups are shown in panels B and E, C and F, and D and G, respectively. The results shown are the mean ± SEM. *P* < 0.05 was considered statistically significant by the Mann-Whitney test.

**FIG 6:**
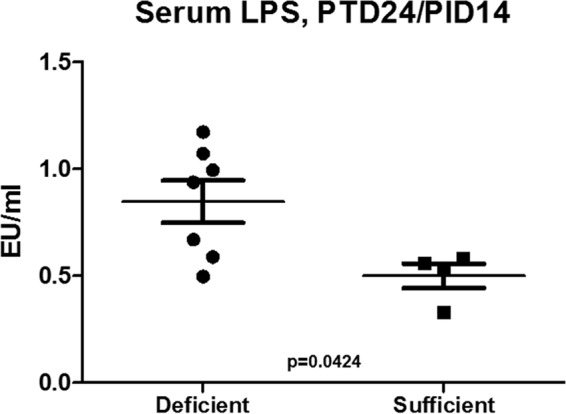
Serum LPS levels of protein-deficient (A to D) and -sufficient (A and E to G) HIFM/HRV-infected pigs. LPS levels in serum samples from protein-deficient and -sufficient HIFM/HRV-infected pigs (*n =* 4 to 7) were determined with HEK-Blue LPS Detection kit 2 (InvivoGen, CA). The results shown are the mean ± SEM. Concentrations were analyzed with the Kruskal-Wallis rank sum test. EU, endotoxin units.

We compared the villus height to crypt depth (VH/CD) ratios of piglets on sufficient and deficient diets with or without HRV infection or HIFM transplantation (Fig. 4). A deficient diet resulted in a decreased villus length in pigs in different groups that was least pronounced in noninfected pigs, suggestive of additive or synergistic effects between the diet and HRV infection in altering the intestinal morphology. Additionally, we observed extensive epithelial vacuolization in nontransplanted compared to HIFM-transplanted pigs, irrespective of the diet (Fig. 4). To further assess the epithelial barrier integrity of pigs in different experimental groups, we compared villin expression by IECs. Consistent with the above, the latter was higher in sufficient pigs and decreased in deficient pigs (Fig. 5). Finally, we measured serum lipopolysaccharide (LPS) levels (at the termination of the experiment) to evaluate the barrier function of the intestinal epithelium. The results demonstrated that serum LPS levels were significantly higher in protein-deficient (HIFM/HRV) pigs (Fig. 6).

### Protein deficiency differentially affects SOX9, CgA, MUC2, villin, and PCNA gene mRNA levels of IECs.

**FIG 7:**
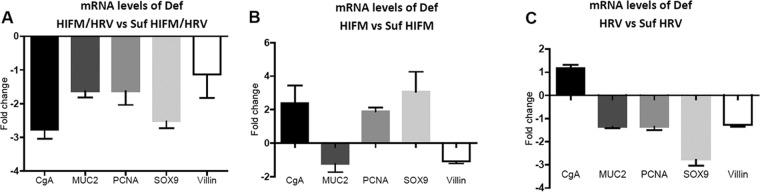
Fold changes in CgA, MUC2, PCNA, SOX9, and villin mRNA levels in IECs of deficient- versus sufficient-diet HIFM/HRV-infected pigs (A), of deficient- versus sufficient-diet HIFM pigs (B), and of deficient- versus sufficient-diet HRV-infected pigs (C) measured by qRT-PCR and normalized to the β-actin gene. The values of sufficient-diet (with or without HIFM, with or without HRV) (A to C) pigs were set to 1. Sufficient- and deficient-diet HRV, *n* = 5 or 6/group; sufficient- and deficient-diet HIFM/HRV, *n* = 10/group; sufficient- and deficient-diet HIFM, *n* = 2 or 3/group. Error bars indicate the SEM.

**FIG 8:**
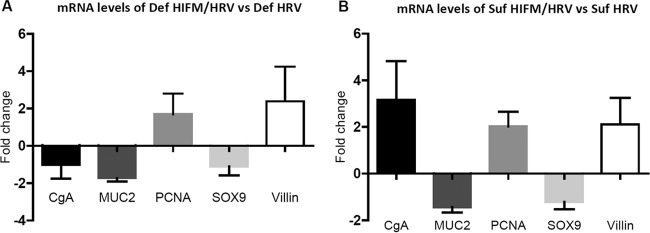
Fold changes in CgA, MUC2, PCNA, SOX9, and villin mRNA levels in IECs of deficient-diet HIFM/HRV-infected versus deficient-diet HRV-infected pigs (A) and in sufficient-diet HIFM/HRV-infected versus sufficient-diet HRV-infected pigs (B) measured by qRT-PCR and normalized to the β-actin gene. The values of deficient-diet HRV-infected (A) and sufficient-diet HRV-infected (B) pigs were set to 1. Sufficient- and deficient-diet HRV-infected pigs, *n* = 5 or 6/group; sufficient- and deficient-diet HIFM/HRV-infected pigs, *n* = 10/group. Error bars indicate the SEM.

SOX9 (expressed by IESCs), MUC2 (expressed by goblet cells), villin (expressed by enterocytes), PCNA (expressed by transient amplifying progenitor cells), and CgA (expressed by enteroendocrine cells) gene mRNA levels were lower in jejunal IECs of protein-deficient pigs than in those of sufficient HIFM/HRV-infected pigs, as demonstrated by quantitative reverse transcription (qRT)-PCR (Fig. 7A). However, only MUC2 and villin mRNA levels were lower in protein-deficient pigs than in protein-sufficient non-HRV-inoculated pigs, while CgA, PCNA, and SOX9 mRNA levels were higher (Fig. 7B). Also, HRV-infected, protein-deficient GF pigs had lower levels of mRNAs of all IEC markers than HRV-infected protein-sufficient pigs, with the exception of CgA mRNA levels, which were higher (Fig. 7C). An additional observation provided by comparison of the mRNA levels of these genes in IECs of HIFM-transplanted and GF pigs inoculated with HRV on protein-deficient and -sufficient diets was that HIFM transplantation resulted in higher villin and PCNA mRNA levels (Fig. 8A and B).

### Protein deficiency results in uniform suppression of several innate immune parameters.

**FIG 9:**
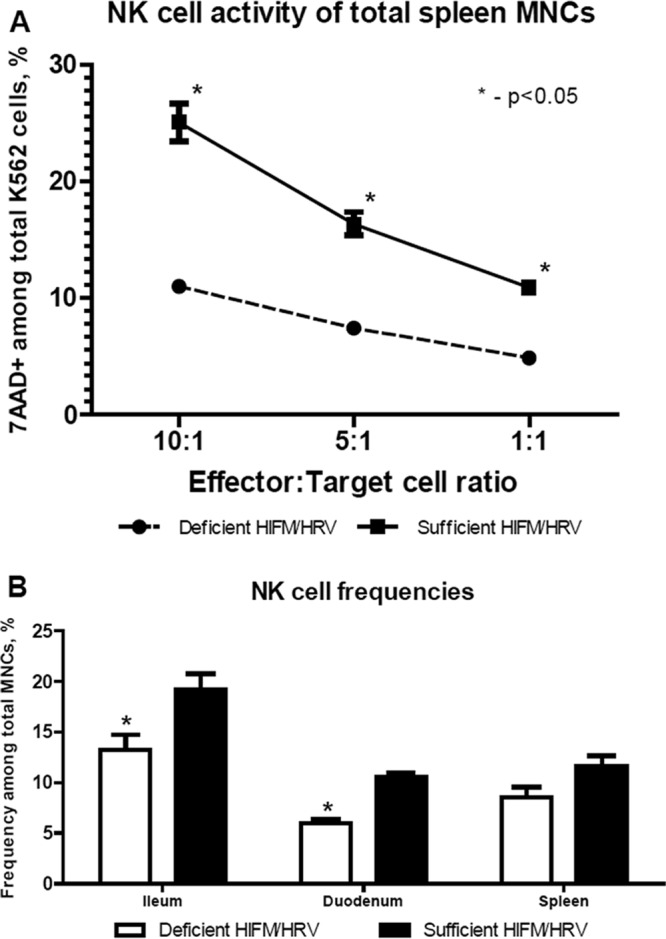
NK cell function of spleen MNCs (A) and NK cell frequencies in the ileum, duodenum, and spleen (B) of protein-deficient and -sufficient HIFM/HRV-infected pigs. MNCs were isolated from the spleen, blood, ileum, and duodenum of piglets in the four experimental groups at PID14. (A) Spleen MNCs (piglets from all experimental groups) and carboxyfluorescein succinimidyl ester-stained K562 tumor cells were used as effector and target cells, respectively, and cocultured at set ratios to assess the cytotoxic function of NK cells (*n* = 10/group). Asterisks indicate statistically significant difference (two-way ANOVA; *P* ≤ 0.05). Error bars indicate the SEM. (B) NK cell frequencies among total MNCs were assessed by flow cytometry (*n* = 10/group). Asterisks indicate statistically significant differences (Kruskal-Wallis test; *P* ≤ 0.05). Error bars indicate the SEM.

**FIG 10:**
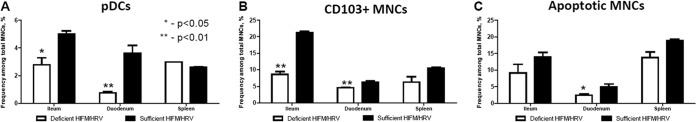
Effects of protein-deficient and -sufficient diets on the frequencies of systemic and intestinal pDCs (A), CD103^+^ MNCs (B), and apoptotic MNCs (C). MNCs were isolated from the spleen, ileum, and duodenum of the piglets in the four experimental groups at PID14. The frequencies of pDCs (CD172^+^/CD4^+^/CD11R1^−^), CD103^+^ MNCs, and apoptotic MNCs among the total MNCs were assessed by flow cytometry. Asterisks indicate statistically significant differences (Kruskal-Wallis test; *P* ≤ 0.05). *n =* 10/group. Error bars indicate the SEM.

**FIG 11:**
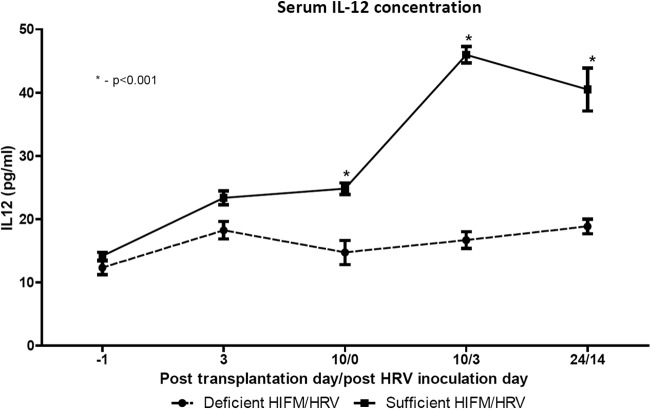
IL-12 cytokine concentrations in serum samples from protein-deficient and -sufficient HIFM/HRV-infected pigs (*n =* 10/group) evaluated with a cytokine ELISA. Asterisks indicate statistically significant differences (two-way ANOVA; *P* < 0.001). Error bars indicate the SEM.

A number of immune parameters (previously identified by us and others as key immune factors in anti-HRV responses) were evaluated at experiment termination (posttransplantation day 28 [PTD28]/PID14) in protein-deficient and -sufficient HIFM/HRV-infected pigs. Protein deficiency decreased the frequencies of natural killer (NK) cells among intestinal (significantly) and splenic (numerically) mononuclear cells (MNCs) and significantly decreased the NK function of splenic MNCs (Fig. 9). Similarly, frequencies of intestinal plasmacytoid dendritic cells (pDCs) and CD103^+^ MNCs were significantly lower in protein-deficient pigs than in protein-sufficient pigs. Finally, the frequencies of apoptotic MNCs were also lower in all of the tissues of protein-deficient pigs (Fig. 10). Although there was a trend for increased levels of most innate cytokines evaluated in serum samples at different time points (data not shown), only IL-12 p40 (referred to as IL-12 throughout) levels were consistently higher in serum samples from protein-sufficient pigs than in those from protein-deficient pigs throughout the duration of the experiment (Fig. 11). No differences in the levels of Toll-like receptor expression by MNCs were noted in any tissues of protein-sufficient or -deficient pigs (data not shown). Similar trends, but without statistically significant differences, were observed in the above immune parameters of control pigs (no HRV or no HIFM).

### Transplantation of microbiota altered by protein malnutrition to Gn pigs on a protein-sufficient diet induces intermediate clinical parameters.

**FIG 12:**
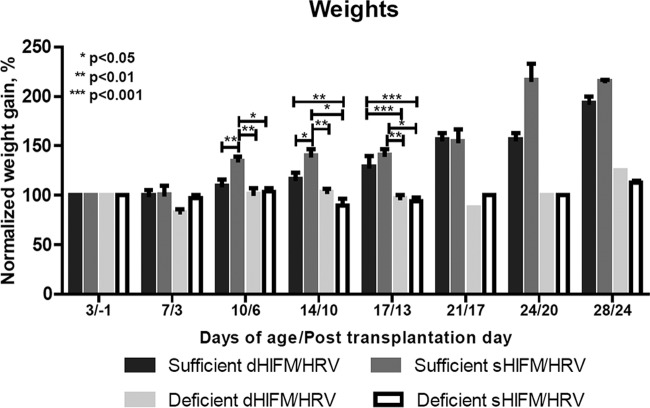
Normalized weight gain of protein-sufficient and -deficient HRV-infected pigs transplanted with sufficient and deficient HIFM (sHIFM/dHIFM) and monitored for 4 weeks. The results are the mean ± SEM from two to four animals of sufficient-diet dHIFM/deficient-diet dHIFM/deficient-diet sHIFM groups, and two or three animals in the sufficient-diet sHIFM group. Statistical analysis was not conducted because of the low animal numbers in some groups.

To evaluate if the malnutrition-associated altered microbiota is sufficient to induce stunting and other clinical parameters associated with malnutrition in a naive host, we transplanted neonatal Gn piglets on the sufficient and deficient diets with HIFM from large intestinal contents of protein-deficient and -sufficient pigs (dHIFM and sHIFM) (Fig. 12). Stunting of the pigs on the deficient diet that were transplanted with sHIFM or dHIFM was indistinguishable from that of pigs transplanted with dHIFM. Likewise sHIFM failed to rescue pigs on a deficient diet from stunting. However, dHIFM pigs on a sufficient diet gained weight more slowly than their sHIFM counterparts, suggesting a negative role for the altered microbiota in the absorption of dietary nutrients and metabolism.

## DISCUSSION

Malnutrition and malnutrition-associated EED contribute to increased childhood deaths from infectious diseases and result in the development of numerous physiological and anatomical deficiencies (1). Protein malnutrition and defective absorption in EED directly affect immune function and the proliferative/regenerative capacity of the intestinal epithelium, as both are highly dependent on protein availability and efficient synthesis (18–23). Subsequently, EED compromises gut morphology and barrier and immune functions, contributing to the systemic spread of intestinal pathogens, commensals, and pathobionts (1, 2, 24). Thus, EED contributes to persistent systemic and intestinal inflammation, further exacerbating malnutrition and intestinal permeability and negatively affecting overall health (1, 5, 25). These alterations of the gut arise early in infancy and inversely correlate with a child’s growth (25–27). Here, we report on the establishment of a neonatal Gn pig model of protein malnutrition—the first HIFM-transplanted large-animal model that most fully recapitulates all of the major aspects of kwashiorkor (protein malnutrition) and EED.

In this study, we reproduced the main clinical parameters associated with protein malnutrition stunting (Fig. 1), hypoproteinemia, hypoalbuminemia, hypoglycemia, and edema (by appearance) in neonatal Gn pigs. Additionally, we demonstrated that these parameters were present but less evident in GF pigs than in HIFM-transplanted protein-deficient pigs, which suggests that altered microbiota associated with protein malnutrition (with increased *Firmicutes* levels and *Firmicutes*-to-*Bacteroidetes* ratios [A. Kumar et al., unpublished data]) could compete with the host for limited dietary resources. Different compositions of the intestinal microbiomes of healthy and malnourished infants and children from developed and developing countries are mostly characterized by decreased microbiome abundance and diversity (or persistent microbiome immaturity), an increased prevalence of “inflammogenic” bacterial taxa and a higher *Firmicutes*-to-*Bacteroidetes* ratio, as well as persistent intestinal colonization by pathogenic/pathobiont bacteria (28). Further, while *Prevotella* was the predominant microbial community signature in healthy children from developing countries (Malawi, Bangladesh, and Venezuela) *Bacteroides* predominated in the North American healthy microbiomes, suggesting geographical and nutritional disparities (29, 30). In this study, we used a fecal transplant from a healthy exclusively breastfed (to minimize dietary variations between developed and developing countries of the respective age group) United States infant to evaluate the role of protein malnutrition in initiating enteric and immune dysfunction, including intestinal dysbiosis. Because protein-deficient HIFM-transplanted pigs with or without HRV were more stunted than protein-deficient GF pigs infected with HRV, we conclude that the altered microbiota and not HRV infection alone plays a major role in the development of stunting in protein deficiency.

In addition to decreased serum protein, albumin, and glucose levels, we observed that protein malnutrition affected the homeostatic control of selenium and vitamins A and E, consistent with similar observations on secondary micronutrient deficiencies associated with childhood malnourishment (1). Thus, unlike in our model of prenatal vitamin A deficiency (single-micronutrient deficiency) (31), protein deficiency affected the levels or homeostatic control of multiple macro- and micronutrients, limiting the ability of neonatal pigs to adapt/maintain homeostasis through compensatory mechanisms. This is in agreement with the uniform suppression of the various innate immune parameters evaluated in this study and associated with protein deficiency versus concurrent up- or downregulation of those in vitamin A-deficient pigs (31).

The increased HRV shedding and severity of diarrhea that we observed in protein-deficient pigs are consistent with most previous observations (1, 24, 32–35). The characteristic second peak of HRV shedding occurs when newly differentiated enterocytes repopulating the small intestinal villi become infected with HRV (36, 37). Thus, the absence of a second shedding peak in the deficient pigs may be suggestive of the inability to replenish the intestinal epithelium with newly generated/migrated enterocytes, potentially reflecting defects in IESC function. In this study, a protein-deficient diet combined with HIFM transplantation and HRV infection resulted in a uniform decrease in the mRNA levels of all of the IEC markers evaluated (CgA, SOX9, PCNA, MUC2, and villin). This, together with decreases in the numbers of IECs expressing the respective markers and villous atrophy (M. A. Alhamo et al., unpublished data), suggests the most profound perturbation of intestinal epithelial homeostasis characterized by inhibition of IEC turnover and regeneration rates, effects consistent with the most severe clinical outcomes observed. In contrast, protein deficiency in HIFM-transplanted noninfected piglets or in HRV-infected GF piglets resulted in decreased MUC2/villin and MUC2/PCNA/SOX9/villin gene mRNA levels, respectively, while the CgA/PCNA/SOX9 and CgA gene mRNA levels were increased. IESCs (SOX9^+^), transient amplifying progenitor cells (PCNA^+^), and enteroendocrine cells (CgA^+^) respond to intestinal insults by enhancing epithelial proliferation and repair. Therefore, the increase in the expression of these respective markers may represent different compensatory mechanisms that are being initiated upon single or dual intestinal insults (protein deficiency plus altered microbiota or protein deficiency plus HRV infection). However, multifactorial insults to the intestinal epithelium (protein deficiency plus HRV plus altered microbiota) likely exhaust its ability to adapt by using alternative biological mechanisms. Finally, our observations suggest that the intestinal microbiota enhances epithelial proliferation and IEC barrier function, which is evident from the higher PCNA and villin mRNA levels in HIFM-transplanted pigs than in GF pigs.

Our previous data and findings by others suggest that NK cells and pDCs are critical for HRV protection, which is consistent with the decreased frequencies of these immune cell subsets and increased HRV diarrhea and shedding (38, 39). Additionally, we demonstrated that optimal NK cell responses against HRV were dependent on efficient IL-12 cytokine production (39). Consistent with that, protein deficiency in this study resulted in decreased NK cell frequencies/function and serum IL-12 levels. CD103^+^ intestinal DCs are necessary to prime and confer intestinal homing of B and T lymphocytes (40). The decrease in CD103^+^ expression on MNCs observed in this study likely contributed to the defective adaptive immune responses of the protein-deficient pigs (D. D. Fischer et al., unpublished data). The frequencies of apoptotic MNCs correlated with the levels of HRV replication in our previous experiments because HRV induces apoptosis of immune and epithelial host cells. Apoptosis of immune cells must be properly regulated to maintain immune homeostasis (41). The decrease in the frequencies of apoptotic MNCs observed in protein-deficient pigs in this study despite higher HRV replication further suggests enhanced activation of survival signaling pathways in MNCs from protein-deficient pigs. For example, in protein-deficient mice, the expression of intestinal cell kinase (a highly conserved serine/threonine protein kinase that potently activates proproliferation and prosurvival pathways of Wnt/β-catenin) was increased while cellular apoptosis decreased in a caspase-dependent manner, providing a protective compensatory mechanism during nutritional stress (42).

Finally, the intermediate weight gain/stunting profile of dHIFM-transplanted pigs on a sufficient diet suggests that a protein-deficient diet is likely a major contributor to the malnourishment pathophysiology and induction/maintenance of intestinal dysbiosis. In addition, the microbiota (dHIFM) also contributes to suboptimal energy harvest/absorption from the diet.

In conclusion, our results indicate that early-life protein malnutrition in an HIFM-transplanted neonatal Gn pig model accurately reproduces the major aspects of protein malnutrition, including clinical signs, suppressed immunity, increased severity of enteric infection, intestinal dysbiosis, and disrupted epithelial barrier and homeostasis. This model can be used to evaluate physiologically relevant interventions and provides an explanation of why dietary interventions alone may lack efficacy in malnourished children.

## MATERIALS AND METHODS

### Virus.

The Gn pig-adapted (passage 25) HRV Wa G1P[8] strain was used to orally inoculate Gn pigs as described previously (43).

### Animal experiments and diets.

All of our animal experiments were approved by the Institutional Animal Care and Use Committee at The Ohio State University (protocol 2010A00000088). All of the pigs were maintained, sampled, and euthanized humanely. Euthanasia was performed by electrocution following anesthesia. For this study, we used the lowest number of pigs previously shown to permit the detection of statistically significant differences among treatments (44, 45). Near-term sows (Landrace × Yorkshire × Duroc cross-bred) were purchased from the Ohio State University Swine Center facility. Caesarean-derived Gn piglets were maintained in sterile isolators as previously described (4). Nursing piglets may consume between 100 and 900 ml of sow milk, which contains 5% protein, 8% fat, and 5% carbohydrates (CHO), per day. To avoid differences in nutrient intake between different groups/litters in this study, the diet intake was restricted throughout experiments. We fed gradually increasing volumes (500 to 800 ml/day) of Parmalat (bovine whole milk containing 3.3% protein, 3.3% fat, and 5% CHO) to piglets in the sufficient-diet group to provide adequate amounts of protein and fat and increased amounts of CHO compared to the daily consumption of sow milk. Similar to the 50% dietary restriction described by Zijlstra et al. (46), piglets in the deficient-diet groups were fed increasing volumes (500 to 800 ml/day) of Parmalat milk mixed with sterile water (50:50, vol/vol), which resulted in a protein content twice as low as that of the sufficient-diet groups. Prior to HIFM transplantation, sterility was verified by aerobic and anaerobic culturing of rectal swabs to ensure that there was no bacterial or fungal contamination. Additionally, all piglets were confirmed to be negative for RV, transmissible gastroenteritis virus, porcine epidemic diarrhea virus, calicivirus/sapovirus, astrovirus, and kobuvirus (47–51). Neonatal piglets from eight litters (12 to 16 pigs/litter) were assigned to different treatment groups (Table 2) to conduct pilot and main experiments.

**TABLE 2:** Pig allocation to experimental groups

Exptl group	Diet	Age (days) at HIFM transplantation	HRV inoculation/euthanasia	*n*
Deficient/HIFM/early HRV	Deficient	4	PTD3/PID5	5
Sufficient/HIFM/early HRV	Sufficient	4		5
Deficient/HIFM/late HRV	Deficient	4	PTD10/PID14	10
Sufficient/HIFM/late HRV	Sufficient	4		10
Deficient/late HRV	Deficient			6
Sufficient/late HRV	Sufficient			5
Deficient/HIFM	Deficient	4	No/PID14	2
Sufficient/HIFM	Sufficient	4		3

The collection and use of infant fecal samples were approved by the Ohio State University Institutional Review Board (protocol 2016H0276). After parental consent was obtained, pooled fecal samples collected from the soiled diapers of a healthy, 2-month-old, exclusively breastfed, vaginally derived United States infant were weighed and diluted 1:20 (wt/vol) in phosphate buffer solution containing 0.05% (vol/vol) cysteine and 30% sterile glycerol as described previously (52). Pigs in the respective groups (Table 2) were orally fed 2 ml of the diluted fecal samples at 4 days of age. Pig weights were measured twice a week with a hand-held digital scale (Brecknell).

At PTD10, pigs were infected with HRV (PID0). The efficiency of HIFM transplantation and the similarity of the microbial compositions of the original infant and Gn pig fecal samples was confirmed by 16S metagenomic (V4-V5 region sequencing) analysis based on open operational taxonomic unit picking by using the GreenGenes 16S rRNA reference database (2013-08 release) as described elsewhere (see Fig. S1 in the supplemental material) (53, 54). Rectal swabs were collected daily (for 8 days) from HRV Wa-inoculated piglets (1 × 10^6^ focus-forming units [FFU]/dose) starting at PID0 to assess HRV shedding and to record fecal scores to assess the severity of diarrhea. The piglets in the early and late HRV groups were euthanized at PID5 and PID14, respectively. Blood, duodenum, ileum, and spleen tissues were collected to isolate MNCs as described previously (55). Serum samples collected at the end of the experiment were submitted to Marshfield Labs (Marshfield, WI) and the Diagnostic Center for Population and Animal Health (Michigan State University, East Lansing, MI) to assess levels of protein/albumin/glucose and vitamin A/vitamin E/selenium, respectively. Additionally, the midsection of the jejunum (10 in. long) was collected to isolate IECs by a modified protocol adapted from reference 56. Briefly, the jejunum was cut into small pieces (1 cm) and placed in a 50-ml tube with 20 ml of Hanks’ balanced salt solution (Gibco BRL, Gaithersburg, MD) containing 5% fetal bovine serum (FBS; Gibco BRL, Gaithersburg, MD) and 2.5 mM EDTA (Sigma-Aldrich, St. Louis, MO). The tissue was agitated twice in an orbital shaker at 300 rpm for 15 min, and the resulting cell suspension was filtered through a metal cell strainer. The IECs were centrifuged at 500 × *g* for 10 min at 4°C, and the pellet was resuspended in RPMI 1640 (Gibco BRL, Gaithersburg, MD) enriched with 8% FBS, 2 mM l-glutamine Gibco BRL, Gaithersburg, MD), 0.1 mM nonessential amino acids (Gibco BRL, Gaithersburg, MD), 1 mM sodium pyruvate (Gibco BRL, Gaithersburg, MD), 20 mM HEPES (Gibco BRL, Gaithersburg, MD), 100 g of gentamicin (VetOne, Boise, ID) per ml, and 10 g of ampicillin (Gibco BRL, Gaithersburg, MD) per ml (E-RPMI). The viability and numbers of IECs were determined by the trypan blue exclusion method. The IECs were stored at −80°C in 500 µl of RNA later tissue collection buffer (Life Technologies, Inc., Carlsbad, CA) until further analysis.

10.1128/mSphere.00046-17.1 FIG S1  Microbiota comparison of the original HIFM sample and HIFM-transplanted pig samples at PTD7. The microbiota shared by the original HIFM and HIFM-transplanted pig feces is green, the unique microbiota is red, and the undetected microbiota is gray. Download FIG S1, PDF file, 0.02 MB. Copyright © 2017 Vlasova et al. 2017 Vlasova et al. This content is distributed under the terms of the Creative Commons Attribution 4.0 International license.

Additionally, one crossover experiment was conducted to evaluate the role of an altered microbiota in the development of clinical parameters of protein malnutrition (stunting/edema). In this experiment, HIFM passaged through protein-deficient pigs (dHIFM) was harvested after 4 weeks on a protein-deficient diet and transplanted into two groups of GF pigs on protein-deficient and -sufficient diets. HIFM passaged through protein-sufficient pigs (sHIFM) served as a control and was similarly transplanted into two groups of GF pigs on protein-deficient and -sufficient diets (*n* = 2 to 4/group).

### Histopathologic analysis and immunofluorescent evaluation of villin expression by IECs.

At necropsy, ileum tissue (5 cm anterior to the ileocecal valve) was collected. After 48 h of fixation in 10% neutral buffered formalin, tissue sections were trimmed, processed, and embedded in paraffin. Four-micrometer-thick sections were cut and stained with hematoxylin and eosin (H&E) or used for immunofluorescent staining. After H&E staining, at least 10 villi and crypts in each section were measured with a computerized image system and VH/CD ratios were calculated as previous described (57). After heat-induced epitope retrieval (60°C, 10 to 15 min), immunofluorescent staining to evaluate villin expression was conducted with an anti-villin goat polyclonal primary antibody (1:500; Santa Cruz, CA) and a donkey anti-goat secondary antibody (1:200; Abcam, Inc., CA). Villin-stained tissues were scored as follows: 0, no villin-positive cells; 1, 1 to 29% villin-positive villous epithelium; 2, 30 to 69% villin-positive villous epithelium; 3, 70 to 100% villin-positive villous epithelium. Reduction of villin-positive epithelium was characterized by lack of or irregular distribution and mildly to moderately reduced expression of villin in villous epithelial cells of the ileal sections. Microscopic images (×20) were obtained with a fluorescence microscope (Olympus IX70-S1F2).

### Evaluation of serum LPS levels.

To measure LPS levels in serum samples from pigs, we used HEK-Blue LPS Detection kit 2 (InvivoGen, CA) according to the manufacturer’s recommendations.

### RNA extraction and qRT-PCR to quantify of CgA, MUC2, PCNA, SOX9, and villin gene mRNA levels.

Total RNA was extracted from IECs with Direct-Zol RNA Miniprep (Zymo Research, Irvine, CA) according to the manufacturer’s instructions. RNA concentration and purity were measured with a NanoDrop 2000c spectrophotometer (Thermo Scientific, Wilmington, DE). Real-time qRT-PCR was performed with equal amounts of total RNA (75 ng) and the Power SYBR green RNA-to-CT 1 step RT-PCR kit (Applied Biosystems, Foster, CA). The primers for CgA, MUC2, PCNA, SOX9, villin, and β-actin were based on previously published data (58–60). Relative CgA, MUC2, PCNA, SOX9, and villin gene expression was normalized to β-actin and expressed as fold change by the 2^−ΔΔ*CT*^ method (61).

### Flow cytometry.

Flow cytometry staining was performed the same day immediately after the isolation of all tissue-derived cells. Procedures for flow cytometry staining to assess the frequencies and distribution of different innate immune cell subsets were as described previously (62). Data acquisition was done with MACSQuant Analyzer 10 (Miltenyi Biotec, Inc.), and analysis was done with FlowJo software (LLC). The pDC-enriched fraction (referred to as pDCs) was defined as described previously (39). Frequencies/tissue distribution of CD103^+^ MNCs were evaluated as previously described (31). The frequencies and tissue distribution of apoptotic MNCs were assessed with annexin V Apoptosis Detection kit APC (eBioscience, San Diego, CA)/propidium iodide staining solution (eBioscience). The analysis and gating strategies were as described previously (39). Additionally, NK cells were defined as CD16^+^ CD172^−^(SWC3a^−^).

### NK cytotoxicity assay.

Total spleen MNCs and K562 tumor cells were used as effector and target cells, respectively. Effector-to-target cell ratios of 10:1, 5:1, 1:1, and 0.5:1 were used, and the assay was done as described previously (63).

### Cytokine ELISA.

The levels of the cytokines porcine alpha interferon, IL-6, tumor necrosis factor alpha, IL-12, and IL-10 in the collected supernatants were examined by enzyme-linked immunosorbent assay (ELISA) with anti-swine cytokine antibodies as previously described (55, 64).

### Statistical analysis.

Two-way analysis of variance (ANOVA-general linear model), followed by Bonferroni’s posttest, was used to compare fecal consistency scores, log-transformed HRV shedding titers, normalized weights, mean levels of the cytokine IL-12, and NK cell activity. The frequencies of cell populations in flow cytometry and serum glucose, protein, albumin, LPS, vitamin A ands E, and selenium levels were compared among groups with the Kruskal-Wallis rank sum and Mann-Whitney (nonparametric) tests. The chi-square/Fisher exact or *t* test (if the mortality rate was 0 in one of the groups) was used to compare the mortality rates of different groups. Statistical significance was assessed at *P* ≤ 0.05 for all comparisons. All statistical analyses were performed with GraphPad Prism 5 (GraphPad Software, Inc., CA).
