# Supplementary material for: Protein Malnutrition Modifies Innate Immunity and Gene Expression by Intestinal Epithelial Cells and Human Rotavirus Infection in Neonatal Gnotobiotic Pigs
Source: mSphere. 2017 Mar 1;2(2):e00046-17. doi: 10.1128/mSphere.00046-17 (PMC5332602; doi:10.1128/mSphere.00046-17)
Supplement: FIG S1 [file sph002172242sf1.pdf]

Assigned OTUs		HIFM	HIFM pig feces
Actinobacteria	*Rothia*		
	Bifidobacterium		
Bacteroidetes	*Rikenellaceae*		
Firmicutes	*Planococcaceae*		
	Ureibacillus		
	Staphylococcus		
	Enterococcus		
	Lactobacillus		
	Streptococcus		
	Clostridiales		
	Clostridiaceae		
	Clostridium		
	SMB53		
	Lachnospiraceae		
	[Ruminococcus]		
	Blautia		
	Peptostreptococcaceae		
	Ruminococcaceae		
	Oscillospira		
	Ruminococcus		
	Megasphaera		
	Veillonella		
Proteobacteria	*Shewanella*		
	Enterobacteriaceae		
	Citrobacter		
	Proteus		
	Trabulsiella		
	Halomonas		
Unassigned			
Abundance of common OTUs (%)		100	99,94
Abundance of unique OTUs (%)		0	0,06
Abundance of unassigned OTUs (%)		0.03	0.17
